# Intravenous Immunoglobulin Reveals a Novel Protective Mechanism: Targeting the GBP5-Driven Pyroptosis Axis in Experimental Colitis

**DOI:** 10.3390/ph19060972

**Published:** 2026-06-22

**Authors:** Qian Long, Tong Wang, Jia He, Xiaochen Yan, Zongkui Wang, Changqing Li, Rong Zhang

**Affiliations:** 1 Institute of Blood Transfusion, Chinese Academy of Medical Sciences & Peking Union Medical College, Chengdu 610052, China; long-qian@scu.edu.cn (Q.L.);; 2 West China School of Public Health and West China Fourth Hospital, Sichuan University, Chengdu 610041, China

**Keywords:** ulcerative colitis, IVIG, pyroptosis, GBP5, NLRP3 inflammasome, DSS model

## Abstract

**Background**: Ulcerative colitis (UC) is a chronic inflammatory bowel disease characterized by mucosal barrier disruption and dysregulated immune responses. While Intravenous Immunoglobulin (IVIG) is widely used for its immunomodulatory effects in various autoimmune conditions, its specific therapeutic mechanisms and molecular targets in colitis remain to be fully elucidated. **Objective**: To elucidate the therapeutic mechanisms of IVIG in dextran sodium sulfate (DSS)-induced colitis, with a focus on pyroptosis regulation via the NOD-like receptor (NLR) signaling pathway. **Methods**: Colitis was induced in mice via DSS administration. IVIG was administered intravenously during disease progression. Colon tissues underwent proteomic profiling, and key targets (GBP5, NLRP3, Pro-Caspase-1, GSDMD) were validated by Western blotting (WB), while interleukin (IL)-1β and IL-18 levels were quantified via ELISA. **Results**: IVIG significantly attenuated weight loss, Disease Activity Index (DAI) scores, colon shortening, and histopathological damage. Proteomics analysis identified 172 differentially expressed proteins between DSS and DSS + IVIG groups, with pronounced downregulation of GBP5 and NLR pathway components. IVIG suppressed GBP5/NLRP3/CASP1 activation, reduced GSDMD cleavage, and significantly decreased IL-1β production (while showing a decreasing trend for IL-18). **Conclusions**: IVIG ameliorates colitis by inhibiting the GBP5/NLRP3/CASP1-mediated pyroptosis pathway, highlighting its potential as a targeted therapy for ulcerative colitis.

## 1. Introduction

Ulcerative colitis (UC) is an inflammatory bowel disease (IBD) characterized by continuous mucosal inflammation of the colon and rectum [1,2]. Its clinical manifestations include diarrhea, abdominal discomfort and bloody stool, with persistent progression, and recurrent inflammation being notable features [3]. UC pathogenesis involves multiple factors, including genetic predisposition, epithelial barrier defects, dysregulated immune responses and microbial dysbiosis [3,4,5]. Specifically, dysregulated colorectal microflora significantly contributes to activating the pyroptotic pathway, initiating an inflammasome- and gasdermin-dependent inflammatory cell death process in UC [6]. This specific cell death mechanism exacerbates the damage to mucosal and epithelial cells, compromising the intestinal barrier and increasing susceptibility to bacterial invasion [7]. Bacterial stimulation of the intestine triggers compensatory immune responses that further exacerbate inflammatory response [7,8]. The interplay between barrier defects and bacterial invasion creates a vicious cycle of inflammation, driving disease progression [9,10,11].While conventional therapies and biologics targeting cytokines or leukocyte migration provide symptom relief, they are often limited by adverse effects, primary non-response, or secondary loss of response [12]. Consequently, there is an urgent need for novel therapeutic strategies that address the root causes of UC pathogenesis.

Intravenous immunoglobulin (IVIG), a plasma-derived product containing pooled IgG, has emerged as a potent immunomodulatory agent for autoimmune and inflammatory diseases [13]. Our previous studies, along with others, have demonstrated that IVIG effectively alleviates symptoms in dextran sulfate sodium (DSS)-induced colitis, restoring colonic length and barrier integrity while suppressing inflammatory cytokines [14,15,16,17,18]. However, while these phenotypic benefits are well-documented, the precise molecular sensors and signaling networks governing IVIG’s protective effects remain poorly defined. Therefore, the novelty of this study lies not in re-validating the efficacy of IVIG, but in dissecting the specific mechanistic depth of its action.

Emerging evidence suggests a link between IVIG and the regulation of the NLR family-mediated immune response. In conditions such as Kawasaki disease and sepsis, IVIG has been shown to downregulate NLRP3 levels and modulate the expression of inflammasome components like NLRC4 and NLRP12 [19,20,21]. Despite these observations in other inflammatory milieus, the specific regulatory role of IVIG on the inflammasome machinery within the context of colitis remains to be fully elucidated.

To bridge this gap, we shifted our focus from phenotypic validation to mechanistic dissection using a quantitative proteomic approach. Among the modulated networks, we identified Guanylate-binding protein 5 (GBP5) as a pivotal target. Unlike other NLR components, GBP5 is a unique interferon-inducible GTPase that acts as a critical upstream sensor and positive regulator of the canonical NLRP3 inflammasome [22]. Mechanistically, GBP5 acts as a molecular checkpoint for inflammasome activation. By interacting with the pyrin domain of NLRP3, it promotes the oligomerization of the adaptor protein ASC, thereby facilitating the secretion of IL-18 and IL-1β and ultimately inducing pyroptosis [23]. We specifically focused on GBP5 because its role as an upstream amplifier makes it a strategic target for controlling the downstream cytokine storm (e.g., IL-1β, IL-18) often observed in colitis, which IVIG is known to suppress [24,25].

In this study, we employed quantitative proteomics, followed by Western blotting and ELISA validation, to map the global protein networks modulated by IVIG. We demonstrate that IVIG may exert its protective effects in colitis by targeting the GBP5-NLRP3 axis, providing new mechanistic insights into IVIG therapy and highlighting GBP5 as a potential therapeutic target for UC.

## 2. Results

### 2.1. IVIG Attenuates Disease Severity in DSS-Induced Colitis

**Figure 1:**
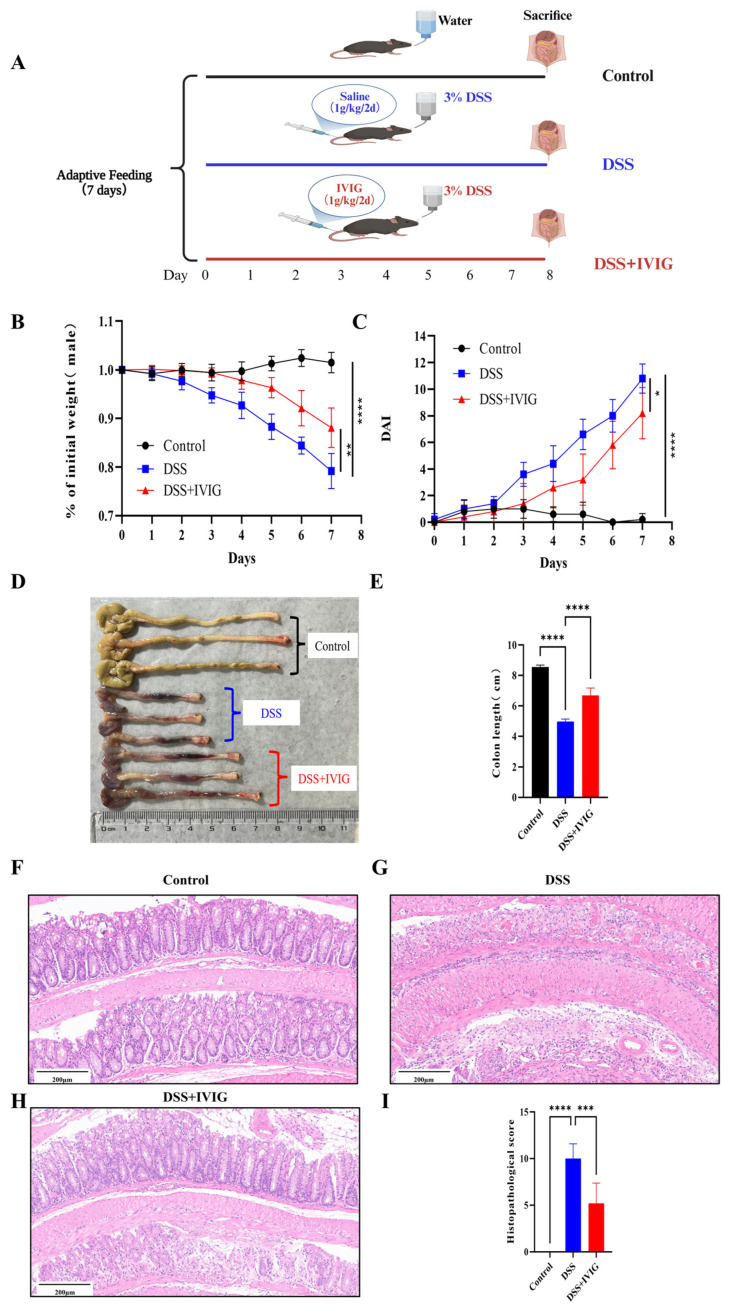
IVIG treatment ameliorates disease severity and pathological damage in DSS-induced colitis. (**A**) Experimental timeline. (**B**) Body weight changes expressed as percentage of initial weight. (**C**) Disease Activity Index (DAI) scores. (**D**) Representative images of colons from each group (dissected on day 8). (**E**) Colon length measurement. (**F**–**H**) Representative H&E-stained colon sections (scale bar, 200 μm). (**I**) Histological scores. Data are presented as mean ± SD (*n* = 5 mice per group). * *p* < 0.05, ** *p* < 0.01, *** *p* < 0.001, **** *p* < 0.0001 (one-way ANOVA with Tukey’s post hoc test).

An acute mouse colitis model induced by DSS was used to study the therapeutic effect of IVIG (Figure 1A). As shown in Figure 1B–E, DSS administration resulted in significant body weight loss (*p* < 0.0001), increased disease activity index (DAI) scores (*p* < 0.0001), and shortened colon length (*p* < 0.0001) compared to control group, confirming successful establishment of the colitis model. Importantly, IVIG treatment markedly attenuated these pathological changes (*p* < 0.05). This substantial improvement in clinical parameters suggests that IVIG exerts potent protective effects against intestinal inflammation, potentially through modulation of immune cell activation and inflammatory mediator production.

Histopathological analysis further revealed that DSS induced severe epithelial damage, crypt loss, and transmural inflammatory cell infiltration, whereas IVIG treatment significantly preserved epithelial integrity and reduced inflammatory infiltration (Figure 1F–I, *p* < 0.001). The preservation of mucosal architecture and reduction in inflammatory cell recruitment indicate that IVIG may enhance epithelial barrier function and suppress the infiltration of pathogenic immune cells into the intestinal mucosa.

### 2.2. Proteomic Profiling Identifies IVIG-Mediated Regulation of NLR Signaling Pathway

**Figure 2:**
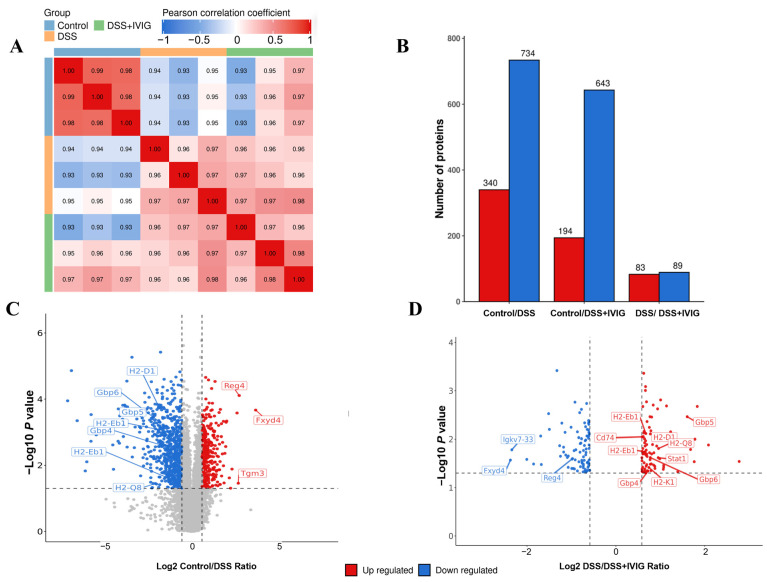
Proteomic profiling reveals distinct expression patterns and IVIG-mediated modulation of protein expression in DSS-induced colitis. (**A**) Pearson’s correlation coefficient (PCC) plot of proteomic data among samples. (**B**) Diagram showing the number of DEPs between groups. (**C**) Volcano plot of DEPs between Control and DSS groups. (**D**) Volcano plot of DEPs between DSS and DSS + IVIG groups. DEPs were defined by |fold change| > 1.5 and *p* < 0.05 (*n* = 3 biologically independent samples).

To characterize the global protein expression alterations induced by DSS and the therapeutic impact of IVIG, we first performed a comprehensive proteomic analysis. The distinct clustering patterns observed in the proteomic landscape (Figure 2A) demonstrate that DSS administration triggers extensive molecular reprogramming, a hallmark of acute colonic inflammation. Subsequent comparative analysis between the DSS and DSS + IVIG groups identified 172 DEPs (83 upregulated, 89 downregulated) based on a cutoff threshold of |fold change| > 1.5 (Figure 2B). Notably, IVIG treatment significantly reversed the dysregulation of key inflammatory mediators. Specifically, IVIG downregulated critical components of the NLR signaling pathway—particularly guanylate-binding proteins (GBPs) such as GBP4, GBP5, and GBP6 (Figure 2C,D). Additionally, molecules involved in antigen presentation, including MHC class II molecules (e.g., H2-Q8, H2-D1), were also downregulated, indicating a potential modulation of antigen presentation processes (Figure 2C,D and Supplementary File S1).

**Figure 3:**
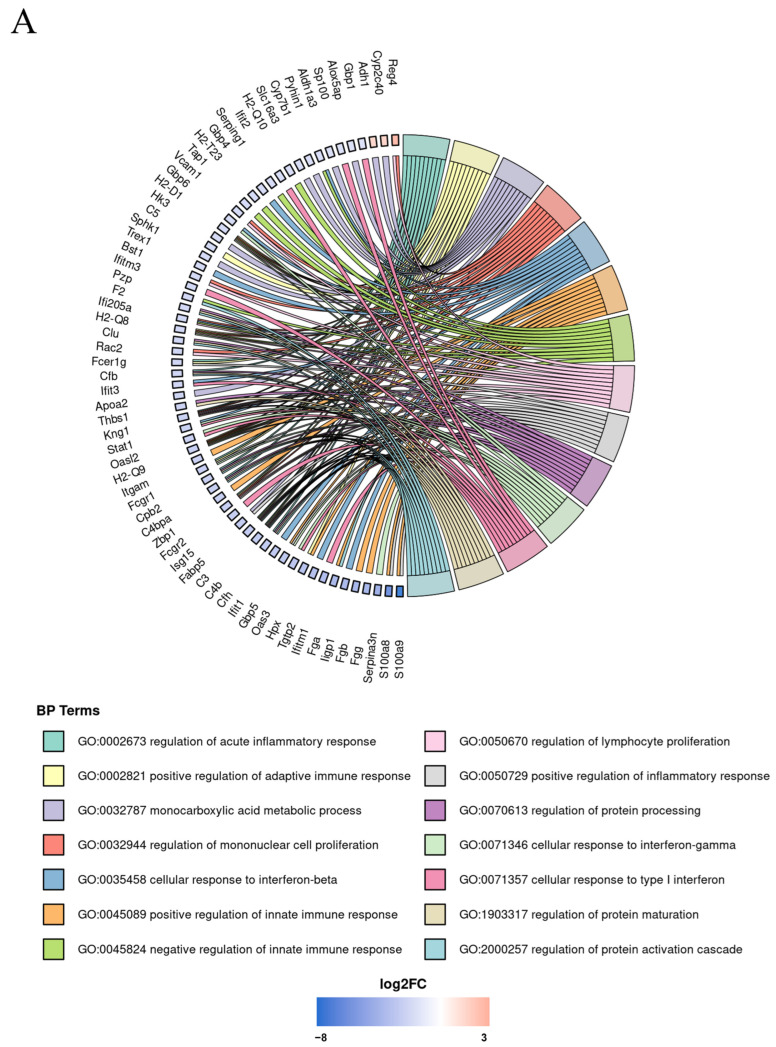

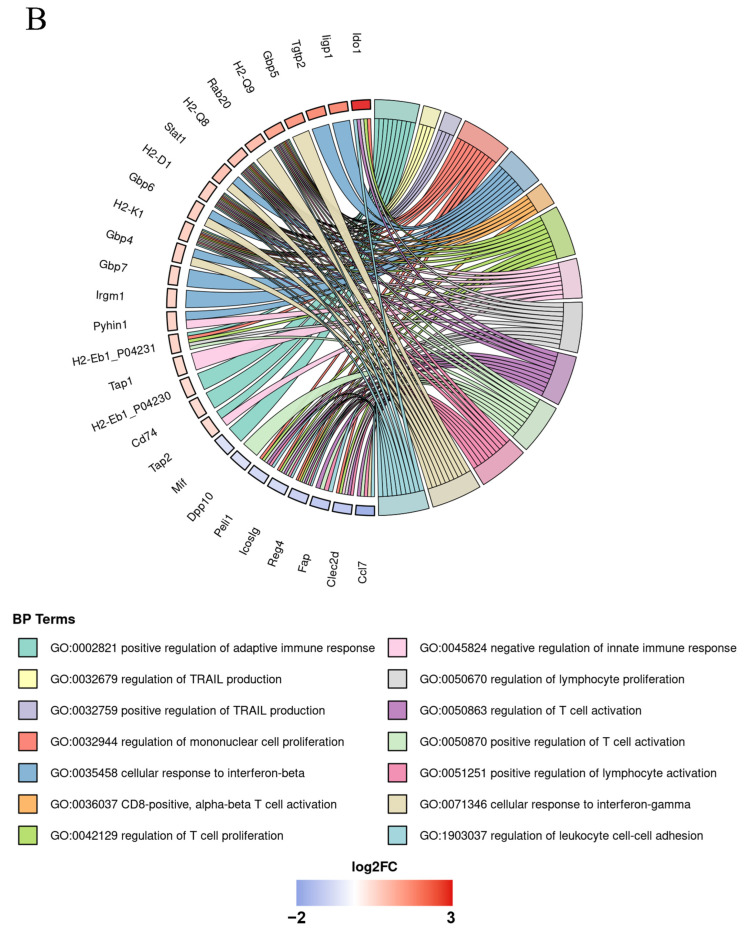
GO enrichment analysis visualizing the associations between DEPs and enriched immune-related biological processes. (**A**) Interactions in the Control vs. DSS comparison reveal DSS-induced inflammatory activation. (**B**) Interactions in the DSS vs. DSS + IVIG comparison highlight the immunomodulatory effects of IVIG treatment. The ribbons connecting genes to terms indicate the involvement of specific genes in corresponding biological functions. The color of the ribbons corresponds to the biological process categories defined in the legend. The color intensity of the gene labels on the arc represents the log^2^ fold change (log^2^FC) of gene expression, with the scale bar shown at the bottom (blue indicates downregulation; red indicates upregulation). Significantly enriched terms (*p* < 0.05) are shown (*n* = 3 biologically independent samples).

To further decipher the biological implications of these DEPs, we performed Gene Ontology (GO) enrichment analysis and visualized the interactions between the DEPs and enriched biological processes using chord diagrams (Figure 3). As illustrated in Figure 3A,B, the vast majority of enriched biological processes were associated with inflammatory responses and immune regulation, underscoring the pivotal roles these pathways play in both colitis progression and IVIG treatment. Corroborating the proteomic findings, proteins linked to these processes—specifically MHC class II molecules (such as H2-Q8, H2-Q9, and H2-Eb1) and the GBP family—were heavily implicated in the enriched pathways. Collectively, these findings suggest that IVIG may ameliorate colitis in mice by modulating MHC class II molecules and the GBP family, thereby attenuating inflammatory responses and restoring immune homeostasis.

**Figure 4:**
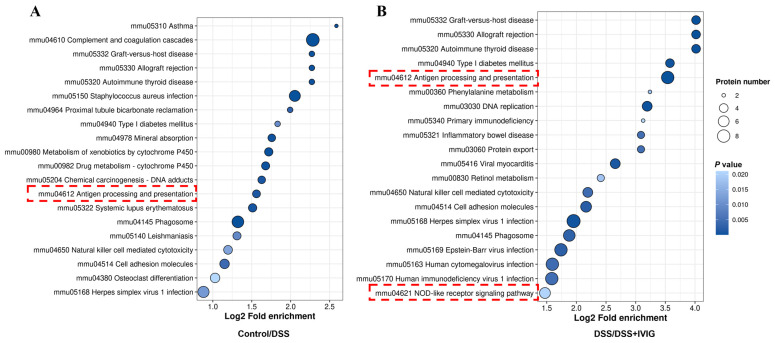
KEGG pathway analysis highlights the NOD-like receptor signaling pathway as a key target of IVIG treatment. (**A**) KEGG pathway enrichment analysis of the DEPs between Control and DSS groups. (**B**) KEGG pathway enrichment analysis of the DEPs between DSS and DSS + IVIG groups. The red dashed boxes indicate the key pathways discussed in the main text. Significantly enriched pathways (*p* < 0.05) are shown (*n* = 3 biologically independent samples).

To further elucidate the specific signaling cascades regulated by IVIG beyond general biological processes, we performed KEGG pathway enrichment analysis (Figure 4). Consistent with the severe inflammatory phenotype induced by DSS, the comparison between Control and DSS groups revealed a hyperactivation of immune-related pathways, most notably “Antigen processing and presentation” (mmu04612) (Figure 4A). This finding corroborates our earlier observation of upregulated MHC molecules, suggesting that aberrant antigen presentation is a primary driver of colitis pathogenesis. Crucially, the therapeutic effect of IVIG appears to be mechanistically linked to the suppression of specific inflammatory axes. As shown in Figure 4B, the “NOD-like receptor signaling pathway” (mmu04621) was identified as one of the most significantly enriched pathways downregulated by IVIG treatment. Given that the previously identified DEPs (specifically the GBP family) are key effector components of this pathway, these data suggest that IVIG may ameliorate colitis by targeting the NOD-like receptor signaling cascade, thereby attenuating the downstream inflammatory response.

**Figure 5:**
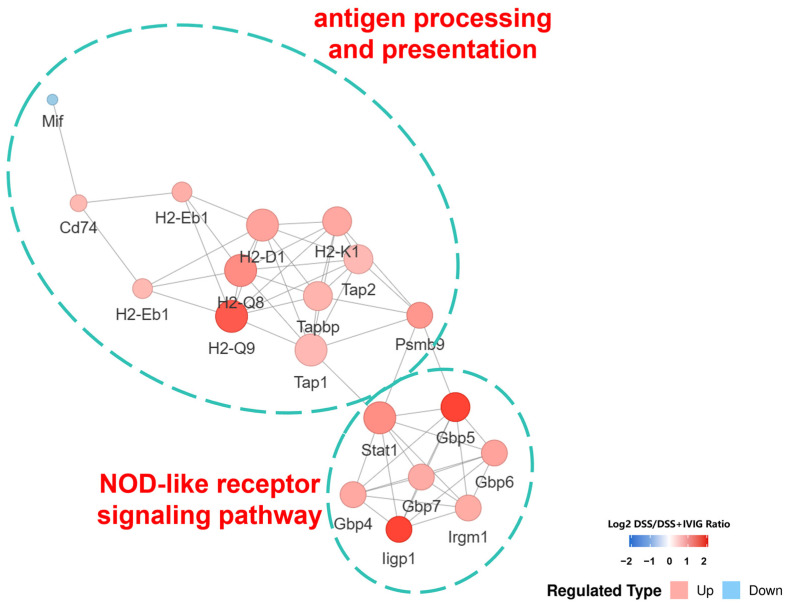
PPI network analysis identifies functional modules centered on MHC molecules and GBPs.

To systematically identify the core regulatory networks modulated by IVIG, we constructed a protein–protein interaction (PPI) network focusing on DEPs (Figure 5). Visual inspection of the network, colored by expression ratio (Log^2^ DSS/DSS + IVIG), revealed a coordinated downregulation of two functionally distinct but interconnected modules.

Specifically, the analysis highlighted a tight cluster of proteins involved in antigen processing and presentation (e.g., H2-Q8, H2-D1) and a central hub associated with the NOD-like receptor signaling pathway (e.g., GBP5, STAT1). The robust interactions observed within and between these clusters suggest that IVIG exerts its therapeutic effects not by targeting isolated proteins, but by simultaneously dampening these synergistic inflammatory axes.

The PPI network of DEPs between DSS and DSS + IVIG groups was constructed using the STRING database and visualized in Cytoscape (Version 3.9.1). Nodes represent DEPs. Red nodes denote proteins upregulated by DSS but downregulated following IVIG treatment, representing potential IVIG-inhibited targets; blue nodes indicate the inverse pattern, highlighting targets restored by IVIG. Color intensity reflects the magnitude of the fold change, while node size is proportional to the number of interacting proteins. The size of the node represents the degree of connectivity. Two key functional clusters, “antigen processing and presentation” and “NOD-like receptor signaling pathway,” are highlighted with dashed circles.

### 2.3. IVIG Suppresses the GBP5/NLRP3/CASP1 Pyroptosis Axis

**Figure 6:**
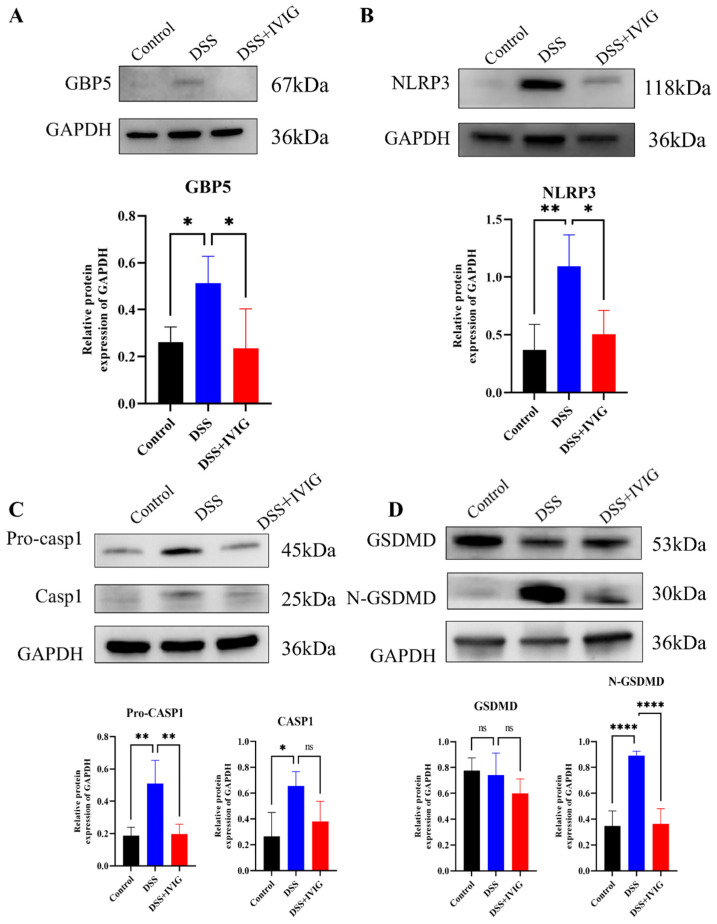
IVIG treatment inhibits the activation of the GBP5/NLRP3/CASP1 pyroptosis axis in colon tissues. (**A**–**D**) Representative Western blot images (upper panels) and quantitative analysis (lower panels) of (**A**) GBP5, (**B**) NLRP3, (**C**) Pro-CASP1 and Cleaved CASP1, and (**D**) GSDMD and cleaved N-GSDMD protein levels in colonic tissues harvested on day 8. GAPDH served as a loading control. Data are presented as mean ± SD (*n* = 4 independent experiments). * *p* < 0.05, ** *p* < 0.01, **** *p* < 0.0001, ns, not significant (one-way ANOVA with Tukey’s post hoc test).

Based on our proteomic findings, we hypothesized that IVIG ameliorates colitis by inhibiting the GBP5/NLRP3/CASP1-mediated pyroptosis pathway. Colonic tissues were harvested on day 8 for protein extraction and subsequent Western blot analysis. The results confirmed that IVIG treatment significantly downregulated key components of this pathway, including GBP5 (Figure 6A, *p* < 0.05), NLRP3 (Figure 6B, *p* < 0.05), and pro-CASP1 (Figure 6C, *p* < 0.01). IVIG also markedly reduced the cleavage of gasdermin D (GSDMD) into its active N-terminal fragment (N-GSDMD) (Figure 6D, *p* < 0.0001). These results indicate that IVIG disrupts the GBP5-NLRP3 axis, thereby preventing the terminal lytic cell death associated with severe colitis.

### 2.4. IVIG Reduces Pyroptosis-Associated Cytokine Production

**Figure 7:**
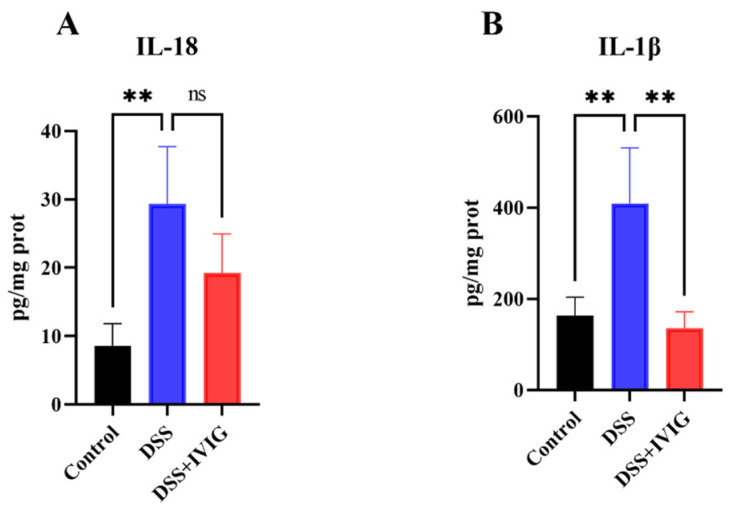
IVIG reduces the levels of pyroptosis-executing cytokine IL-1β and shows a trend towards decreasing IL-18 (**A**,**B**) ELISA of (**A**) IL-1β and (**B**) IL-18 levels in colon tissues. Data are presented as mean ± SD (*n* = 4 mice per group). ** *p* < 0.01, ns, not significant (one-way ANOVA with Tukey’s post hoc test).

ELISA quantification of colonic cytokines revealed that IVIG treatment significantly decreased IL-1β levels (Figure 7A, *p* < 0.01) compared to the DSS group. Regarding IL-18, although the difference was not statistically significant, the IVIG-treated group exhibited a downward trend compared to the DSS group (Figure 7B, *p* > 0.05). These findings suggest that IVIG effectively suppresses the NLRP3 inflammasome downstream effector IL-1β, and may potentially attenuate IL-18 production in this study.

### 2.5. Experimental Validation of Proteomic Findings by qRT-PCR

**Figure 8:**
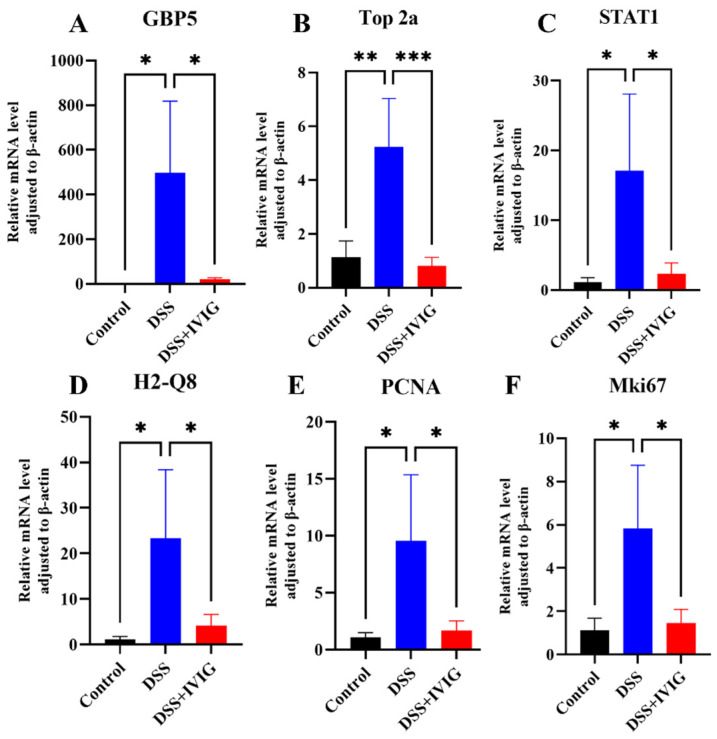
qRT-PCR validation confirms IVIG-mediated downregulation of key differentially expressed genes identified by proteomics. (**A**–**F**) mRNA expression levels of (**A**) GBP5, (**B**) Top2a, (**C**) STAT1, (**D**) H2-Q8, (**E**) PCNA, and (**F**) Mki67 were measured by quantitative RT-PCR. Data are presented as mean ± SD (*n* = 4 mice per group). * *p* < 0.05, ** *p* < 0.01, *** *p* < 0.001 (one-way ANOVA with Tukey’s post hoc test).

Finally, quantitative RT-PCR analysis was performed to confirm the transcriptional regulation of key targets identified in the proteomic screen. IVIG treatment significantly downregulated the mRNA expression of GBP5, STAT1, and the antigen presentation molecule H2-Q8 (Figure 8A–D, all *p* < 0.05). Consistent with the resolution of inflammation, IVIG also significantly reduced the expression of proliferation markers PCNA and Mki67 (both *p* < 0.05). This suggests that IVIG treatment alleviates the inflammation-driven epithelial hyperproliferation, thereby promoting the restoration of colonic tissue homeostasis.

## 3. Discussion

IBD, particularly UC, is characterized by intestinal inflammation and immune dysregulation [1,4]. IVIG has emerged as a promising therapeutic agent due to its broad immunomodulatory and anti-inflammatory properties [26]. Consistent with our previous finding [18], this study demonstrates that IVIG administration significantly alleviates the clinical symptoms and histological damage in a DSS-induced colitis model. Our results confirm that IVIG treatment leads to a reduction in DAI scores and ameliorates colon shortening, aligning with the established efficacy of IVIG in experimental colitis [14,15].

The identification of the specific molecular targets mediating IVIG’s effects remains a critical challenge. In the present study, we employed a multi-step approach to elucidate the underlying mechanism. Initially, proteomic analysis identified GBP5 as a potential key target, exhibiting a significant alteration following IVIG therapy. To validate this finding, we performed WB and ELISAs. These experiments confirmed that IVIG treatment significantly downregulated the protein expression of GBP5 and its downstream effector NLRP3. Furthermore, we observed a marked reduction in the cleavage of N-GSDMD and a concomitant decrease in the levels of the pyroptosis-associated cytokine IL-1β in colonic tissues. Collectively, these findings suggest that the downregulation of the GBP5/NLRP3 axis constitutes a pivotal event in the therapeutic mechanism of IVIG.

Mechanistically, the NLRP3 inflammasome plays a central role in innate immunity and inflammatory responses [27]. Upon activation, NLRP3 recruits pro-Caspase-1, leading to its autocatalytic cleavage and the subsequent processing of pro-inflammatory cytokines IL-1β and IL-18 [27,28]. Furthermore, Caspase-1 cleaves Gasdermin D (GSDMD), releasing its N-terminal fragment to form plasma membrane pores, which is the hallmark of pyroptosis. Our data indicate that IVIG treatment suppresses this cascade, as evidenced by the reduced levels of cleaved Caspase-1 and GSDMD observed in our validation experiments. This suggests that IVIG mitigates intestinal inflammation, at least in part, by inhibiting the GBP5-dependent pyroptosis pathway.

While our data place GBP5 as a critical node, how IVIG administration leads to GBP5 downregulation remains to be defined. It is worth noting that the downregulation of GBP5 following IVIG treatment is not merely a secondary consequence of general inflammation alleviation, but rather a specific molecular event potentially driven by IVIG’s direct modulation of the interferon signaling pathway. IVIG binding to cell-surface Fcγ receptors (FcγRs), particularly the inhibitory FcγRIIb, can trigger anti-inflammatory signaling cascades that block the activation of key transcription factors such as STAT1 [29]. In line with this, our qRT-PCR data (Figure 8C) show that IVIG significantly reduces STAT1 mRNA levels. Given that STAT1 is a master transcriptional driver of many interferon-stimulated genes—including the GBP family, downstream suppression of STAT1 would be expected to lower GBP5 transcription. In addition, IVIG can be internalized into cells via endocytosis, particularly through interactions with Fc receptors (FcγRs) or the neonatal Fc receptor (FcRn) [30,31]. Once internalized, IVIG (or its bioactive components) could directly influence intracellular signaling environments.

Several limitations of this study should be acknowledged. First, while the DSS-induced colitis model is widely used, it does not fully recapitulate the complex pathophysiology of human UC. Second, and most importantly, our data show a strong association between IVIG treatment, reduced GBP5 expression, and attenuated pyroptosis, but we have not established causality. Definitive proof would require genetic loss-of-function models (e.g., *Gbp5*-knockout mice) or pharmacological inhibition of NLRP3. Such validation was beyond the scope of the present work and remains a critical direction for future studies. Third, it remains unclear which cell type (e.g., macrophages, epithelial cells, or infiltrating immune cells) drives GBP5 modulation following IVIG treatment, limiting our understanding of its cell-type-specific actions in the gut. Fourth, our proposed upstream signaling mechanisms (e.g., via FcγRIIb and STAT1) are hypothetical and await direct experimental testing. Finally, the translational relevance of our findings to human UC needs to be confirmed using patient samples or public transcriptomic datasets.

## 4. Materials and Method

### 4.1. Experimental Mice

All animal procedures were approved by the Ethics Committee of Chinese Academy of Medical Sciences, Institute of Blood Transfusion, Chinese Academy of Medical Sciences & Peking Union Medical College (Approval No. 2022034). Staff were trained in animal care and handling.

Specific pathogen-free (SPF) grade male C57BL/6 mice, weighing 22–25 g (corresponding to 6–7 weeks of age), were procured from GemPharmatech Co., Ltd. (Nanjing, China; Approval No. SCXK (Chuan) 2020-0034). Upon arrival, the animals were acclimatized for 7 days under standard housing conditions (temperature: 18–23 °C; humidity: 40–60%; 12 h light/dark cycle) with free access to standard chow and water.

### 4.2. DSS-Induced Colitis Model and Treatment Regimen

Colitis was induced using dextran sulfate sodium (DSS; MW 36,000–50,000; MP Biomedicals, Irvine, CA, USA) following established protocols. Briefly, mice received 3.0% (*w*/*v*) DSS dissolved in sterile drinking water for 7 consecutive days. This concentration was selected based on previous literature and preliminary experiment to induce moderate colitis suitable for therapeutic intervention studies [32].

IVIG has been used in two distinct dose regimes: low-dose (0.4–0.8 g/kg) replacement therapy in primary immunodeficient patients and high-dose (1–2 g/kg) in autoimmune and inflammatory diseases [33]. Based on published literature and our pilot dose-ranging studies, an IVIG dose of 1 g/kg was selected to mimic the high-dose regimen used clinically for autoimmune and inflammatory diseases, thereby ensuring the translational relevance of our findings [14,34]. From day 1 of DSS induction, mice received IVIG (Shandong Taibang Biological Products Co., Ltd., Tai’an, China, 201710S01, 1 g/kg) via tail vein injection every 48 h until sacrifice on day 8.

### 4.3. Group Allocation

Animals were randomly allocated into three experimental groups using a random number generator: (1) Control group (*n* =20): Received standard drinking water; (2) DSS group (*n* = 20): Received 3.0% DSS in drinking water and saline injections (1 g/kg/2 d); (3) DSS + IVIG group (*n* = 20): Received 3.0% DSS in drinking water and IVIG injections (1 g/kg/2 d).

For endpoint analyses, a subset of tissue samples was used for specific assays due to tissue availability. Specifically, *n* = 5 mice per group were used for histological scoring (H&E), *n* = 3 mice per group were used for proteomic analysis, while *n* = 4 biological replicates per group were utilized for WB, qRT-PCR, and ELISA analyses. All investigators involved in data collection and analysis were blinded to the group allocation.

### 4.4. Disease Activity Index (DAI)

The severity of colitis was monitored daily by assessing the Disease Activity Index (DAI). The DAI score (ranging from 0 to 12) was calculated as the sum of scores for body weight loss (0–4), stool characteristics (0–4), and hematochezia (0–4), as previously described [35].

### 4.5. Tissue Collection and Processing

At the experimental endpoint (Day 8), mice were anesthetized with isoflurane and euthanized via cervical dislocation. The colon was excised from the cecum to the anus. The length of the colon was measured as a macroscopic indicator of inflammation. Tissue segments were snap-frozen in liquid nitrogen for molecular analysis or fixed in 4% paraformaldehyde for histology.

### 4.6. Histopathological Evaluation

Paraformaldehyde-fixed colon tissues were embedded in paraffin, sectioned (4 μm thickness), and stained with Hematoxylin and Eosin (H&E). Histological scoring was performed in a blinded manner based on five parameters: ulcer depth (0–4), ulcer extent (0–4), inflammation severity (0–4), inflammation extent (0–4), and fibrosis location (0–4). Total score range: 0–20 [36]. The investigator was blinded to the group allocation during scoring.

### 4.7. Protein Extraction and Western Blotting (WB)

Total protein was extracted from colon tissues using RIPA lysis buffer supplemented with protease inhibitors (RIPA lysis buffer and protease inhibitors, Servicebio, G2033-100ML and G2008-1ML, Wuhan, China). Protein concentration was determined using the BCA Protein Assay Kit (Thermo Fisher Scientific, 23227, Waltham, MA, USA). Equal amounts of protein were separated on 12% SDS-PAGE gels and transferred onto polyvinylidene difluoride (PVDF) membrane. After blocking with 5% non-fat milk, membranes were incubated overnight at 4 °C with primary antibodies against GBP5 (Abcam, EPR28367-83, Waltham, MA, USA), Caspase-1 (MedChemexpress, HYP80622, Monmouth Junction, NJ, USA), NLRP3 (HUABIO, ET1610-93, Huangzhou, China), and GSDMD (HUABIO, HA721144, Huangzhou, China). GAPDH was used as a loading control.

After incubation with HRP-conjugated secondary antibodies, protein bands were visualized using enhanced chemiluminescence (ECL) reagents. Images were captured using the Cytiva Amersham ImageQuant 500 (Cytiva, Marlborough, MA, USA) and quantified using Image J software (Version 1.54g 18 October 2023). The investigator was blinded to the group allocation during scoring.

### 4.8. Quantitative RT-PCR (qRT-PCR)

Total RNA was isolated from colon tissues using the TransZol Up Plus RNA Kit (TransGene Biotech, ER501-01-V2, Beijing, China). cDNA was synthesized using the TranScript All-in-One First-Strand cDNA Synthesis SuperMix (TransGene Biotech, AE341-02, Beijing, China). qRT-PCR was performed using PerfectStart™ Green qPCR SuperMix (TransGene Biotech, AQ202-01, Beijing, China) on a CFX96 Touch Real-Time PCR Detection System (Version 2.3).

The target genes analyzed included pro-inflammatory cytokines and inflammasome components: GBP5, Top 2a, STAT1, H2-Q8, PCNA and Mki67. All qPCR primers are listed in Supplementary File S4. The relative mRNA expression levels were calculated using the 2^−ΔΔCt^ method, with β-actin serving as the endogenous reference gene [37].

### 4.9. Proteomic Analysis

#### 4.9.1. Sample Preparation and Trypsin Digestion

Colon tissues were ground in liquid nitrogen. Proteins were extracted with lysis buffer (8 M urea, 1% protease inhibitor cocktail), sonicated, and centrifuged (12,000 *g*, 4 °C, 10 min). Protein concentration was determined via BCA assay.

Proteins precipitated in acetone (−20 °C, 2 h), redissolved in 200 mM TEAB. Digested overnight with trypsin (1:50 ratio). Reduced with 5 mM DTT (56 °C, 30 min), alkylated with 11 mM iodoacetamide (RT, 15 min), and desalted via SPE columns.

#### 4.9.2. LC-MS/MS Analysis

The list of DEPs with a fold change > 1.5 and *p* < 0.05 was used as the input for subsequent bioinformatic analyses. To generate this dataset, liquid Chromatography-Tandem Mass Spectrometry (LC-MS/MS) was performed. The resulting peptides were analyzed on a timsTOF Pro mass spectrometer (Bruker, Bremen, Germany) coupled with an Easy-nLC 1000 system. The mass spectrometry proteomics data have been deposited to the ProteomeXchange Consortium via the PRIDE [38] partner repository with the dataset identifier PXD068491.

#### 4.9.3. Bioinformatics Analysis

The identified proteins served as the input dataset for bioinformatics analysis.

Gene Ontology (GO) and KEGG Pathway Analysis: Functional enrichment analysis was performed using the KEGG database (http://www.genome.jp/kegg/kaas/, accessed on 9 April 2024) and GO database (v5.0.2, http://eggnog5.embl.de/#/app/home/, accessed on 9 April 2024). The GO analysis focused on Biological Process (BP).

Protein–Protein Interaction (PPI) Network: To predict functional interactions among DEPs, the STRING database (https://cn.string-db.org/, accessed on 9 April 2024) was utilized. The analysis was restricted to Mus musculus (mouse). we applied a confidence score threshold of >0.7. The resulting interaction network was subsequently imported into the R environment (4.3.0) and rendered using the “visNetwork” package (2.1.4) to generate interactive visualizations.

### 4.10. Enzyme-Linked Immunosorbent Assay (ELISA)

To evaluate the local inflammatory response, colon tissue homogenates were prepared. Protein samples were processed as described in Section 4.7. The concentrations of IL-1β and IL-18 in the supernatants were measured using commercial ELISA kits according to the manufacturers’ instructions (Mouse IL-1 Beta ELISA Kit: Boster, Pleasanton, CA, USA, EK0394; Mouse IL-18 ELISA Kit: Sangon Biotech, Shanghai, China, D721113).

### 4.11. Statistical Analysis

Data are presented as mean ± standard deviation (SD). Statistical significance between multiple groups was determined using one-way analysis of variance (ANOVA) followed by Tukey’s post hoc test for multiple comparisons. A value of *p* < 0.05 was considered statistically significant. All analyses were performed using JASP (version 0.96.0.0).

## 5. Conclusions

**Figure 9:**
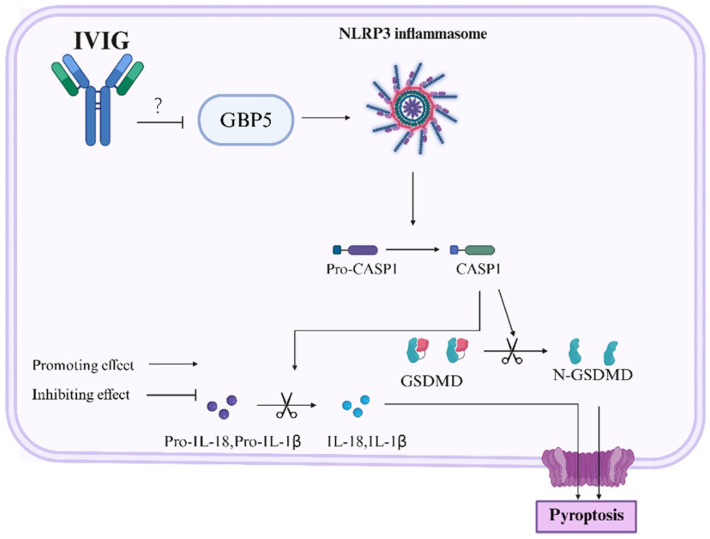
A proposed model illustrating the mechanism by which IVIG alleviates DSS-induced colitis via inhibition of the GBP5/NLRP3/CASP1 axis. Schematic diagram summarizing the proposed mechanism. DSS-induced colitis activates the GBP5/NLRP3 inflammasome, leading to CASP1 activation, GSDMD cleavage, pyroptosis, and release of pro-inflammatory cytokines (IL-1β/IL-18). IVIG treatment inhibits GBP5 expression, thereby suppressing the entire pyroptosis signaling cascade and alleviating intestinal inflammation. “?” indicates an undefined step in the mechanism.

In summary, our findings provide novel insights into the mechanism of IVIG in treating experimental colitis. By integrating proteomic profiling with wet-lab validation, we highlighted the GBP5/NLRP3/CASP1 axis as a key target of IVIG. As shown in Figure 9, this study suggests that IVIG may exert its therapeutic effects by disrupting the GBP5-mediated activation of the NLRP3 inflammasome, thereby suppressing pyroptosis and alleviating intestinal inflammation.

## Data Availability

The data presented in this study are openly available in the PRIDE database (http://www.ebi.ac.uk/pride, accessed on 9 April 2024), reference number PXD068491.

## Supplementary Materials

The following supporting information can be downloaded at: https://www.mdpi.com/article/10.3390/ph19060972/s1, Supplementary File S1: Detailed information of differentially expressed proteins among experimental groups; Supplementary File S2: Detailed information of Go terms and its related proteins among experimental groups; Supplementary File S3: Detailed information of KEGG pathway and its related proteins among experimental groups; Supplementary File S4: Detailed information of primer sequence; ARRIVE checklist.

## References

[1] Kobayashi T., Siegmund B., Berre C.L., Chen W.S., Marc F., Shen B., Bernstein C.N., Danese S., Peyrin-Biroulet L., Hibi T. (2020). Ulcerative colitis. Nat. Rev. Dis. Prim..

[2] Le Berre C., Honap S., Peyrin-Biroulet L. (2023). Ulcerative colitis. Lancet.

[3] Ungaro R., Mehandru S., Allen P.B., Peyrin-Biroulet L., Colombel J.F. (2017). Ulcerative colitis. Lancet.

[4] Ramos G.P., Papadakis K.A. (2019). Mechanisms of Disease: Inflammatory Bowel Diseases. Mayo Clin. Proc..

[5] Du L., Ha C. (2020). Epidemiology and Pathogenesis of Ulcerative Colitis. Gastroenterol. Clin..

[6] Cruz H., Shah P., Wohlgemuth N., Ketchum R., Nassif I., Hu C.A. (2025). Pyroptosis in ulcerative colitis: Biomarkers and therapeutic targets. J. Biomed. Sci..

[7] Chen Y., Cui W., Li X., Yang H. (2021). Interaction Between Commensal Bacteria, Immune Response and the Intestinal Barrier in Inflammatory Bowel Disease. Front. Immunol..

[8] Suzuki T. (2020). Regulation of the intestinal barrier by nutrients: The role of tight junctions. Anim. Sci. J..

[9] Maloy K.J., Powrie F. (2011). Intestinal homeostasis and its breakdown in inflammatory bowel disease. Nature.

[10] Kang S., Kim J., Park A., Koh M., Shin W., Park G., Lee T.A., Kim H.J., Han H., Kim Y. (2023). TRIM40 is a pathogenic driver of inflammatory bowel disease subverting intestinal barrier integrity. Nat. Commun..

[11] Ahn J., Son S., Oliveira S.C., Barber G.N. (2017). STING-Dependent Signaling Underlies IL-10 Controlled Inflammatory Colitis. Cell Rep..

[12] Liu Y., Li B.G., Su Y.H., Zhao R.X., Song P., Li H., Cui X.H., Gao H.M., Zhai R.X., Fu X.J. (2022). Potential activity of Traditional Chinese Medicine against Ulcerative colitis: A review. J. Ethnopharmacol..

[13] Martínez T., Garcia-Robledo J.E., Plata I., Urbano M.-A., Posso-Osorio I., Rios-Serna L.J., Barrera M.C., Tobón G.J. (2019). Mechanisms of action and historical facts on the use of intravenous immunoglobulins in systemic lupus erythematosus. Autoimmun. Rev..

[14] Kozicky L.K., Menzies S.C., Hotte N., Madsen K.L., Sly L.M. (2019). Intravenous immunoglobulin (IVIg) or IVIg-treated macrophages reduce DSS-induced colitis by inducing macrophage IL-10 production. Eur. J. Immunol..

[15] Charlet R., Sendid B., Kaveri S., Poulain D., Bayry J., Jawhara S. (2019). Intravenous Immunoglobulin Therapy Eliminates *Candida albicans* and Maintains Intestinal Homeostasis in a Murine Model of Dextran Sulfate Sodium-Induced Colitis. Int. J. Mol. Sci..

[16] He J., Jiang P., Ma L., Liu F. (2024). Intravenous immunoglobulin protects the integrity of the intestinal epithelial barrier and inhibits ferroptosis induced by radiation exposure by activating the mTOR pathway. Int. Immunopharmacol..

[17] Qin Y., Yuan X., Zhang W., Wang Z., Li C. (2023). Therapeutic effects of IVIG, on oxaliplatin-induced spleen and intestinal injuries in mice. Chin. J. Blood Transfus..

[18] Long Q., Wang Z., Li C., Zhang R. (2025). Therapeutic efficacy of intravenous immunoglobulin in ulcerative colitis. Chin. J. Blood Transfus..

[19] Li M., Liu D., Cheng Z., Zhou X., Chen Z., Liu R., Yi Q. (2024). Serum NLRP3: A potential marker for identifying high-risk coronary arterial aneurysm in children with Kawasaki disease. Cytokine.

[20] Yu B., Zheng B., Shen Y., Shen Y., Qiu H., Wu L., Chen Y., Cai X., Wu J., Hong Q. (2024). NLRC4 methylation and its response to intravenous immunoglobulin therapy in Kawasaki disease: A case control study. BMC Pediatr..

[21] Huang Y.H., Lo M.H., Cai X.Y., Kuo H.C. (2018). Epigenetic hypomethylation and upregulation of NLRC4 and NLRP12 in Kawasaki disease. Oncotarget.

[22] Li Z., Qu X., Liu X., Huan C., Wang H., Zhao Z., Yang X., Hua S., Zhang W. (2020). GBP5 Is an Interferon-Induced Inhibitor of Respiratory Syncytial Virus. J. Virol..

[23] Shenoy A.R., Wellington D.A., Kumar P., Kassa H., Booth C.J., Cresswell P., MacMicking J.D. (2012). GBP5 promotes NLRP3 inflammasome assembly and immunity in mammals. Science.

[24] Fann D.Y., Lee S.Y., Manzanero S., Tang S.C., Gelderblom M., Chunduri P., Bernreuther C., Glatzel M., Cheng Y.L., Thundyil J. (2013). Intravenous immunoglobulin suppresses NLRP1 and NLRP3 inflammasome-mediated neuronal death in ischemic stroke. Cell Death Dis..

[25] Fann D.Y., Lim Y.A., Cheng Y.L., Lok K.Z., Chunduri P., Baik S.H., Drummond G.R., Dheen S.T., Sobey C.G., Jo D.G. (2018). Evidence that NF-κB and MAPK Signaling Promotes NLRP Inflammasome Activation in Neurons Following Ischemic Stroke. Mol. Neurobiol..

[26] Galeotti C., Kaveri S.V., Bayry J. (2017). IVIG-mediated effector functions in autoimmune and inflammatory diseases. Int. Immunol..

[27] Huang Y., Xu W., Zhou R. (2021). NLRP3 inflammasome activation and cell death. Cell. Mol. Immunol..

[28] Ranson N., Veldhuis M., Mitchell B., Fanning S., Cook A.L., Kunde D., Eri R. (2018). NLRP3-Dependent and -Independent Processing of Interleukin (IL)-1β in Active Ulcerative Colitis. Int. J. Mol. Sci..

[29] Jones A.T., Marino A.E., Martynyuk T., Bournazos S., Ravetch J.V. (2025). The anti-inflammatory activity of IgG is enhanced by co-engagement of type I and II Fc receptors. Science.

[30] Bayry J., Ahmed E.A., Toscano-Rivero D., Vonniessen N., Genest G., Cohen C.G., Dembele M., Kaveri S.V., Mazer B.D. (2023). Intravenous Immunoglobulin: Mechanism of Action in Autoimmune and Inflammatory Conditions. J. Allergy Clin. Immunol. Pract..

[31] Norris P.A.A., Kaur G., Lazarus A.H. (2020). New insights into IVIg mechanisms and alternatives in autoimmune and inflammatory diseases. Curr. Opin. Hematol..

[32] Jiang X., Chen X., Dong R., Wang J., Pan Y., Cao Y. (2023). Establishment of a mouse model of inflammatory bowel disease using dextran sulfate sodium. Adv. Clin. Exp. Med..

[33] Perez E.E., Orange J.S., Bonilla F., Chinen J., Chinn I.K., Dorsey M., El-Gamal Y., Harville T.O., Hossny E., Mazer B. (2017). Update on the use of immunoglobulin in human disease: A review of evidence. J. Allergy Clin. Immunol..

[34] Qian L. (2025). The Effects and Mechanisms of Intravenous Immunoglobulin in DSS-Induced Murine Colitis. Master’s Thesis.

[35] Zhang J., Lei H., Hu X., Dong W. (2020). Hesperetin ameliorates DSS-induced colitis by maintaining the epithelial barrier via blocking RIPK3/MLKL necroptosis signaling. Eur. J. Pharmacol..

[36] Rachmilewitz D., Karmeli F., Takabayashi K., Hayashi T., Leider-Trejo L., Lee J., Leoni L.M., Raz E. (2002). Immunostimulatory DNA ameliorates experimental and spontaneous murine colitis. Gastroenterology.

[37] Livak K.J., Schmittgen T.D. (2001). Analysis of relative gene expression data using real-time quantitative PCR and the 2^−ΔΔCT^ Method. Methods.

[38] Perez-Riverol Y., Bandla C., Kundu D.J., Kamatchinathan S., Bai J., Hewapathirana S., John N.S., Prakash A., Walzer M., Wang S. (2025). The PRIDE database at 20 years: 2025 update. Nucleic Acids Res..

